# Better Sleep Better Psychological Well‐Being Among Older Adults: Evidence From the Chinese Longitudinal Healthy Longevity Survey (CLHLS)

**DOI:** 10.1155/jare/1858442

**Published:** 2026-07-29

**Authors:** Yunyun Huang, Lizheng Ge, Chun Chen, Songjia Zhang, Rujia Zhang, Xiaoyi Wang, Qingren Yang, Xiangyang Zhang, Xuelan Zhao, Jiajia Liu

**Affiliations:** ^1^ School of Humanities and Management Sciences, Wenzhou Medical University, Wenzhou 325035, China, wmu.edu.cn; ^2^ School of Public Health, Fudan University, Shanghai 200032, China, fudan.edu.cn; ^3^ Institute for County Chronic Disease Health Management Research, Wenzhou Medical University, Taizhou 317600, China, wmu.edu.cn; ^4^ International Business College, Shandong Jiaotong University, Weihai 264200, China, sdjtu.edu.cn; ^5^ Purchasing Department, First Affiliated Hospital of Wenzhou Medical University, Wenzhou 325035, China, wzhospital.cn; ^6^ Department of Geriatrics, Huashan Hospital, National Clinical Research Center for Aging and Medicine, Fudan University, Shanghai 200040, China, fudan.edu.cn

**Keywords:** latent growth model (LGM), longitudinal association, psychological well-being, sleep quality

## Abstract

**Background:**

Older adults’ psychological well‐being is recognized as a public health issue, and many factors may influence it. However, the longitudinal association between sleep quality and psychological well‐being among older adults remains insufficiently understood. Therefore, this study aimed to examine the longitudinal association between sleep quality and psychological well‐being among Chinese older adults.

**Methods:**

This study involved 2371 older adults aged 65 years and above who participated in all four waves (2008, 2011, 2014, and 2018) of the Chinese Longitudinal Healthy Longevity Survey (CLHLS). A latent growth model (LGM) was used to examine the longitudinal association between sleep quality and psychological well‐being among older adults.

**Results:**

An unconditional quadratic growth curve model showed the best model fit. After controlling for time‐invariant and time‐varying covariates, better sleep quality was consistently associated with better psychological well‐being among older adults (2008: *β* = 0.159, *p* < 0.001; 2011: *β* = 0.193, *p* < 0.001; 2014: *β* = 0.207, *p* < 0.001; 2018: *β* = 0.266, *p* < 0.001).

**Conclusion:**

This study found that better sleep quality was longitudinally associated with better psychological well‐being among Chinese older adults over time. The results suggest that enhanced sleep quality among older adults in China is strongly associated with improved psychological well‐being.

## 1. Introduction

Mental health is a major public health concern with substantial impacts on individuals, families, and society [1]. It has been incorporated into the United Nations Sustainable Development Goals framework, highlighting its importance for sustainable development and population well‐being [2]. Rather than merely the absence of mental disorders, mental health reflects a positive state of psychological well‐being. Mental health conditions remain highly prevalent worldwide, affecting approximately one in eight people globally [3]. Older adults are particularly vulnerable, with around 14.1% of individuals aged 70 years and older experiencing a mental disorder [4]. In China, mental health among older adults has become an increasingly important public health issue [5]. Recent evidence suggests that depressive symptoms remain highly prevalent among older adults in China, with prevalence estimates ranging from 30% to 38% in community‐dwelling older adults [6]. Mental disorders also contribute substantially to the global burden of disease, accounting for approximately 155.4 million disability‐adjusted life years worldwide in 2021 [7]. In addition, the economic burden of mental disorders is considerable, with estimated average annual societal costs ranging from $1180 to $18,313 per treated individual [8].

Psychological well‐being is widely recognized as a key dimension of positive mental health, reflecting individuals’ subjective emotional experiences and overall psychological functioning [9, 10]. Maintaining psychological well‐being is particularly important, as it is closely linked to quality of life, functional independence, and healthy aging [11]. However, aging is often accompanied by declines in psychological well‐being due to physical deterioration, chronic disease, social isolation, and bereavement [12]. Identifying modifiable determinants of psychological well‐being is therefore critical for promoting healthy aging.

Sleep problems have also emerged as an important public health issue and are particularly common among older adults [13]. Globally, nearly one‐third of the population experiences sleep‐related health problems [14]. In China, more than 40% of older adults report sleep difficulties [15]. Furthermore, poor sleep quality is more common among older adults (9.7%) compared with younger populations (4.2%), highlighting age‐related disparity in sleep health [15].

Numerous factors, including physical function, chronic disease, social support, nutritional status, and environmental exposures, have been associated with psychological well‐being in later life [16–20]. Among these, sleep quality has emerged as a potentially modifiable factor and has been consistently associated with psychological well‐being [21]. However, evidence regarding their longitudinal relationship remains limited, particularly among Chinese older adults. Although previous studies have applied fixed‐effects models [22], these approaches may not fully capture time‐varying changes [23]. Therefore, a latent growth model (LGM) was employed in this study to examine the longitudinal association between sleep quality and psychological well‐being while accounting for both time‐invariant and time‐varying covariates.

Improving psychological well‐being is a major goal of healthy aging. Compared with many psychological interventions that require substantial resources, sleep represents a potentially modifiable, accessible, and scalable target for intervention [24–26]. Therefore, understanding the longitudinal association between sleep quality and psychological well‐being may provide important evidence for developing practical strategies to promote healthy aging.

Therefore, the aims of this study were to describe the trajectories of sleep quality and psychological well‐being among older adults in China and to examine their longitudinal association from 2008 to 2018.

## 2. Method

### 2.1. Data Sources

The data employed in this study were obtained from the Chinese Longitudinal Healthy Longevity Survey (CLHLS), which is a follow‐up survey on the factors affecting health among older adults in China. The CLHLS database has investigated the basic characteristics, health status, lifestyle, emotional status, and activities of daily living among older adults aged over 65 years and their adult children aged 35–64 years in China since 1998, covering 23 provinces across the country. The enumerators of the database were professionally trained, and face‐to‐face surveys were used to collect data.

**FIGURE 1:**
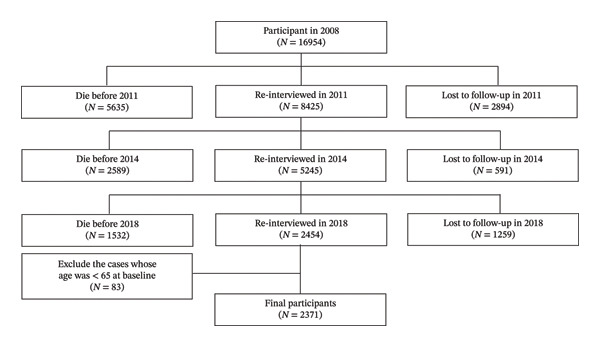
Data screening process from 2008 to 2018.

Four waves of CLHLS data (2008, 2011, 2014, and 2018) were included in this study. A total of 16,954 respondents participated in the 2008 baseline survey. Among them, 2454 respondents completed all four survey waves. Because this study focused specifically on older adults, only participants aged 65 years and above at baseline were eligible for inclusion. Therefore, 83 respondents younger than 65 years at baseline were excluded from the analysis. Finally, 2371 older adults were included in this study (Figure 1).

To assess the potential impact of sample attrition, baseline characteristics between participants retained in the final analytic sample and those lost to follow‐up were compared using *t*‐tests and chi‐square tests, as appropriate. The comparison results are presented in Supporting Table S1.

### 2.2. Measurement

#### 2.2.1. Psychological Well‐Being

Psychological well‐being was assessed using seven psychological well‐being items derived from the psychological status module of the CLHLS. These items have been widely used in previous CLHLS‐based studies to assess psychological well‐being among Chinese older adults and showed acceptable reliability and validity [27, 28]. The seven items assessed the following dimensions of psychological functioning: positive attitudes toward life (“looking on the bright side of things”), personal orderliness (“keeping belongings neat and clean”), negative emotions (“feeling fearful or anxious”), loneliness (“feeling lonely and isolated”), autonomy (“making one’s own decisions”), self‐worth (“feeling useless with age”), and subjective happiness (“feeling as happy as younger people”). Together, these items reflect multiple domains of mental health and psychological well‐being among older adults.

Each item was scored on a five‐point Likert scale ranging from 1 (*always*) to 5 (*never*). Items reflecting positive psychological well‐being status, including looking on the bright side of things, keeping belongings neat and clean, making one’s own decisions, and feeling as happy as younger people, were reverse coded to ensure that higher scores consistently represented better psychological well‐being. The total psychological well‐being score ranged from 7 to 35, with higher scores indicating better psychological well‐being among older adults. The response option “unable to answer” was treated as missing because previous CLHLS studies have suggested that this category is commonly used for respondents with severe physical weakness or cognitive impairment, rather than representing a valid midpoint response [28]. To maintain conceptual consistency and comparability of the composite psychological well‐being measure, respondents with missing data on any of the seven items were initially treated as having missing psychological well‐being scores. Across the four survey waves, 737 participants (31.08%) had incomplete responses on at least one psychological well‐being item. Considering the potential risk of selection bias caused by item‐level missingness, multiple imputation using Bayesian estimation was subsequently conducted to reduce potential bias under the missing‐at‐random assumption.

#### 2.2.2. Sleep Quality

Sleep quality is generally considered a multidimensional construct involving sleep duration, sleep latency, sleep continuity, sleep disturbance, daytime dysfunction, and subjective sleep satisfaction [29]. Standardized multidimensional instruments such as the Pittsburgh Sleep Quality Index (PSQI) are commonly used in clinical sleep research [30]. However, the CLHLS does not include comprehensive sleep assessment tools such as the PSQI across all survey waves. Therefore, consistent with previous population‐based studies using CLHLS data and other large epidemiological surveys [31, 32], this study used a single‐item self‐rated sleep quality measure to represent overall subjective sleep quality among older adults. Subjective sleep evaluations are widely used in large epidemiological studies because they reflect individuals’ overall perceptions and experiences of sleep, which may integrate multiple dimensions of sleep health into a global assessment. In addition, brief sleep assessment measures may be particularly suitable for very old populations because lengthy questionnaires can increase respondent burden, fatigue, and missing data and reduce data quality among older adults with cognitive or physical limitations [33, 34]. Therefore, short subjective sleep indicators may improve feasibility in large longitudinal epidemiological surveys.

Participants were asked: “How about the quality of your sleep?” In the original CLHLS questionnaire, sleep quality was measured on a five‐point ordinal scale, ranging from 1 = *very good*, 2 = *good*, 3 = *fair*, 4 = *poor*, and 5 = *very poor*. This coding reflects worsening sleep quality with increasing scores in the original dataset. To ensure consistency in the direction of interpretation across variables, the sleep quality variable was recoded in this study so that higher values indicated better sleep quality. Specifically, the variable was reverse coded as follows: 1 = *very poor*, 2 = *poor*, 3 = *fair*, 4 = *good*, and 5 = *very good*. This transformation was performed solely for interpretability and comparability in regression and LGMs and did not alter the underlying ordinal structure of the variable. Additionally, the “unable to answer” option was marked as missing.

#### 2.2.3. Covariates

According to previous studies [35, 36], the covariates included time‐invariant and time‐varying variables. Time‐invariant variables included gender (0 = *female*, 1 = *male*) and education (0 = *illiterate*, 1 = *primary school and above*).

Time‐varying variables were divided into social structural and health‐related behavioral factors. Social structural factors were as follows: age, marital status (0 = *unmarried*, 1 = *married*), residence (1 = *city*, 2 = *town*, 3 = *rural*), housing status (0 = *living alone*, 1 = *living with family*), and economic status (1 = *very poor*, 2 = *poor*, 3 = *fair*, 4 = *good*, and 5 = *very good*). Health‐related behavioral factors were as follows: currently exercising (0 = *no*, 1 = *yes*), currently smoking (0 = *no*, 1 = *yes*), currently drinking alcohol (0 = *no*, 1 = *yes*), basic activities of daily living (BADL) disability (0 = *no*, 1 = *yes*), instrumental activities of daily living (IADL) disability (0 = *no*, 1 = *yes*), and chronic diseases status (0 = *no*, 1 = *yes*). Both BADL (6 items) and IADL (8 items) were taken from the international scales used in CLHLS. As long as one of the six items or 8 items cannot be able to do, it is recorded as “having BADL disability” or “having IADL disability.” Time‐invariant variables were taken from the 2008 wave, and time‐varying variables from the current waves of 2008, 2011, 2014, and 2018.

### 2.3. Statistical Analysis

Descriptive statistics were first analyzed for each variable using SPSS 26.0. Subsequently, a LGM was estimated using Mplus 8.3. LGM are longitudinal structural equation models that can estimate trajectories of repeated measures over time while accounting for interindividual variability [37]. In this study, the intercept represented the baseline level of psychological well‐being, whereas the slope represented the rate of change over time. [27]. Considering that a small number of imputations (e.g., *m* = 5) may lead to unstable standard error estimates, we increased the number of imputations to 30 in accordance with previous methodological recommendations suggesting 20–40 imputations for datasets with moderate levels of missing data [38, 39]. All variables included in the analytical model were incorporated into the imputation procedure to ensure congeniality between the imputation and analysis models. Parameter estimates were pooled across imputed datasets using Rubin’s rules [40]. After imputation, LGMs were estimated separately within each imputed dataset, and the final parameter estimates and standard errors were combined across imputations.

The LGMs in this study were constructed to progressively examine the longitudinal association between sleep quality and psychological well‐being in four steps, as follows: (1) An unconditional linear model (Model A), an unconditional linear free score model (Model B), and a quadratic model (Model C) were chosen to explore the growth trajectory of the scores of psychological well‐being. (2) A univariate LGM (Model D) that only incorporated sleep quality and psychological well‐being but did not incorporate other covariates. (3) An LGM incorporating the remaining covariates (Model E), except for sleep quality. (4) An LGM involving all covariates (Model F), including sleep quality. The staged modeling strategy was intended to examine whether the longitudinal association between sleep quality and psychological well‐being remained after adjustment for potential confounding variables.

### 2.4. Sensitivity Analysis

Previous studies noted that the “unable to answer” option in the CLHLS was planned for older adults who were too weak to respond to the self‐rated question [41]. Therefore, the robustness of the results was evaluated using the following three methods for performing sensitivity analysis. First, a complete‐case analysis (CCA) was additionally conducted after excluding all cases with missing values on any variables. Second, sensitivity analysis A was performed after dropping cases with “unable to answer” for psychological well‐being. Third, sensitivity analysis B was performed after dropping cases with “unable to answer” for psychological well‐being and sleep quality.

Additionally, previous studies revealed that a subgroup may have different results, thereby raising doubts about the reliability of the outcomes [42]. Therefore, three sensitivity analyses for the gender, education, and age variables were conducted to reduce the potential influence of reverse causation. The three subgroups of the LGMs were as follows: (a) the gender subgroup (men and women); (b) the education subgroup (illiterate and primary school or above); and (c) the age subgroup (≥ 80 years and < 80 but ≥ 65 years).

## 3. Results

### 3.1. Basic Characteristics

**FIGURE 2:**
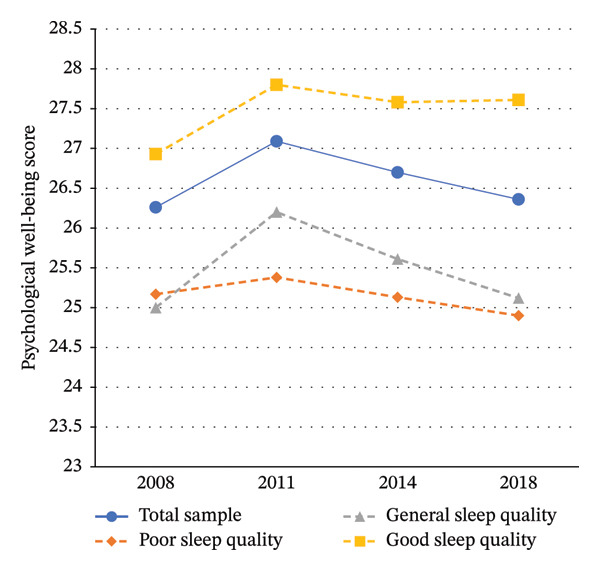
Psychological well‐being changes over time.

A total of 2371 older adults who completed all four survey waves (2008, 2011, 2014, and 2018) were included in this study (Table 1). The older adults comprised 1280 women (53.99%) and 1091 men (46.01%), with a mean baseline age of 75.83 ± 8.11. The average psychological well‐being score for older adults was 26.26 ± 3.73 in 2008, 27.09 ± 3.89 in 2011, 26.70 ± 3.93 in 2014, and 26.36 ± 4.02 in 2018. The score rose abruptly in 2011, after which it declined annually with age (Figure 2). In terms of sleep quality, the percentage of older adults who considered that they had poor or very poor sleep quality raised from 11.4 in 2008 to 14.7 in 2018, whereas the percentage who considered that they had good or very good sleep quality decreased from 64.3 in 2008 to 45.6 in 2018.

**TABLE 1:** Descriptive statistics of full sample and subgroups.

	2008 (%)	2011 (%)	2014 (%)	2018 (%)
Age	75.83 ± 8.11	78.92 ± 8.13	81.77 ± 8.11	85.73 ± 8.04

*Gender*				
Woman	1280 (53.99)	1280 (53.99)	1280 (53.99)	1280 (53.99)
Man	1091 (46.01)	1091 (46.01)	1091 (46.01)	1091 (46.01)

*Education*				
Illiteracy	1161 (48.97)	1161 (48.97)	1161 (48.97)	1161 (48.97)
Primary school or above	1205 (50.82)	1205 (50.82)	1205 (50.82)	1205 (50.82)
Missing	5 (0.21)	5 (0.21)	5 (0.21)	5 (0.21)

*Marital status*				
Unmarried or widowed	938 (39.56)	1057 (44.58)	1164 (49.09)	1327 (55.97)
Married	1433 (60.44)	1301 (54.87)	1158 (48.84)	960 (40.49)
Missing	0 (0)	13 (0.55)	49 (2.07)	84 (3.54)

*Residence*				
Urban	304 (12.82)	372 (15.69)	372 (15.69)	422 (17.80)
Town	477 (20.12)	807 (34.04)	860 (36.27)	924 (38.97)
Rural	1590 (67.06)	1192 (50.27)	1139 (48.04)	1025 (43.23)

*Living with family member*				
No	391 (16.49)	483 (20.37)	539 (22.73)	473 (19.95)
Yes	1980 (83.51)	1864 (78.62)	1799 (75.88)	1756 (74.06)
Missing	0 (0)	24 (1.01)	33 (1.39)	142 (5.99)

*Income*				
Very poor	56 (2.36)	49 (2.07)	43 (1.81)	23 (0.97)
Poor	324 (13.67)	313 (13.20)	207 (8.73)	210 (8.86)
Fair	1689 (71.24)	1572 (66.30)	1691 (71.32)	1567 (66.09)
Good	273 (11.51)	382 (16.11)	355 (14.97)	423 (17.84)
Very good	24 (1.01)	40 (1.69)	35 (1.48)	62 (2.61)
Missing	5 (0.21)	15 (0.63)	40 (1.69)	86 (3.63)

*Sleeping quality*				
Very poor	23 (0.97)	29 (1.22)	31 (1.31)	51 (2.15)
Poor	247 (10.42)	296 (12.48)	287 (12.10)	296 (12.48)
Fair	574 (24.21)	570 (24.04)	684 (28.85)	699 (29.48)
Good	1167 (49.22)	997 (42.05)	944 (39.81)	709 (29.90)
Very good	358 (15.10)	475 (20.03)	394 (16.62)	372 (15.69)
Missing	2 (0.08)	4 (0.17)	31 (1.31)	244 (10.29)

*Physical activity*				
No	1540 (64.95)	1312 (55.34)	1424 (60.06)	1525 (64.32)
Yes	831 (35.05)	1027 (43.32)	874 (36.86)	728 (30.70)
Missing	0 (0)	32 (1.35)	73 (3.08)	118 (4.98)

*Drinking*				
No	1863 (78.57)	1856 (78.28)	1919 (80.94)	1934 (81.57)
Yes	508 (21.43)	479 (20.20)	412 (17.38)	327 (13.79)
Missing	0 (0)	36 (1.52)	40 (1.69)	110 (4.64)

*Smoking*				
No	1850 (78.03)	1894 (79.88)	1921 (81.02)	1927 (81.27)
Yes	521 (21.97)	459 (19.36)	419 (17.67)	357 (15.06)
Missing	0 (0)	18 (0.76)	31 (1.31)	87 (3.67)

*ADL disability*				
No	2314 (97.60)	2148 (90.59)	2039 (86.00)	1714 (72.29)
Yes	57 (2.40)	167 (7.04)	238 (10.04)	529 (22.31)
Missing	0 (0)	56 (2.36)	94 (3.96)	128 (5.40)

*IADL disability*				
No	1624 (68.49)	1383 (58.33)	1150 (48.50)	726 (30.62)
Yes	747 (31.51)	978 (41.25)	1181 (49.81)	1580 (66.64)
Missing	0 (0)	10 (0.42)	40 (1.69)	65 (2.74)

*Chronic disease*				
0	1026 (43.27)	710 (29.95)	773 (32.60)	590 (24.88)
1	1304 (55.00)	1409 (59.43)	1469 (61.96)	1509 (63.64)
Missing	41 (1.73)	252 (10.63)	129 (5.44)	272 (11.47)
Psychological well‐being	26.26 ± 3.73	27.09 ± 3.89	26.70 ± 3.93	26.36 ± 4.02

Supporting Table S1 presents the baseline comparison between participants included in the analytic sample and those lost to follow‐up. Compared with the attrition sample, participants retained in the analytic sample were generally younger and healthier at baseline, with better sleep quality, higher psychological well‐being scores, lower levels of ADL/IADL disability, and lower prevalence of chronic disease (all *p* < 0.001). The analytic sample also included a higher proportion of participants who were married, physically active, and living in rural areas. In addition, the attrition sample showed relatively high missing proportions for baseline mental health (21.16%) and chronic disease status (26.18%), suggesting that participants lost to follow‐up may have had poorer or more unstable baseline health conditions. These attrition patterns are consistent with selective survival and healthy survivor effects commonly reported in longitudinal studies involving older population [43, 44].

### 3.2. Results of the LGM

#### 3.2.1. Model Selection

Model selection was based on trends in the mean of the psychological well‐being scores in the four waves. There were three possible models to choose from: the unconditional linear growth model (Model A), the unconditional linear growth model with free time scores (Model B), and the unconditional quadratic growth curve model (Model C) (Table 2). After checking the fit indices of the three models, in general, the quadratic growth curve model had higher CFI and TLI (CFI = 0.953, TLI = 0.921), and it had lower *χ* ^2^ */df,* RMSEA, and SRMR (*χ* ^2^ */df* = 5.784, RMSEA = 0.045, SRMR = 0.045). Finally, we selected Model C as the optimal model.

**TABLE 2:** Model fit information and comparisons.

Model	Number of parameters	Chi‐square test of model fit	df	*p* value	AIC	BIC	RMSEA (90% CI)	CFI	TLI	SRMR
Model A	8	90.216	6	< 0.001	51954.049	52005.985	0.085 (0.070–0.101)	0.874	0.849	0.044
Model B	10	28.544	4	< 0.001	51868.685	51932.162	0.060 (0.041–0.081)	0.963	0.927	0.055
Model C	12	14.432	2	0.008	51870.160	51945.178	0.075 (0.044–0.112)	0.980	0.881	0.019
Model D	17	74.722	13	< 0.001	50720.665	50818.766	0.045 (0.035–0.055)	0.953	0.921	0.045
Model E	63	385.370	135	< 0.001	50514.093	50877.643	0.028 (0.025–0.031)	0.886	0.840	0.013
Model F	67	382.180	147	< 0.001	50002.470	50407.103	0.026 (0.023–0.029)	0.909	0.873	0.012

*Note:* Model A was the unconditional linear LGM; Model B was the unconditional linear LGM with free time scores; Model C was the unconditional quadratic LGM. Model D was the univariate LGM only with subjective sleep quality; Model E was the LGM with other covariates except subjective sleep quality; Model F was the LGM with subjective sleep quality and other covariates.

Abbreviations: AIC = Akaike information criterion, BIC = Bayesian information criterion, CFI = comparative fit index, LGM = latent growth model, RMSEA = root mean square error of approximation, SRMR = standardized root mean square residual, TLI = Tucker–Lewis index.

#### 3.2.2. Univariate LGM

The results showed that better sleep quality was significantly associated with better psychological well‐being in the unadjusted model (Model D) (2008: *β* = 0.228, 95% CI: 0.192–0.265, *p* < 0.001; 2011: *β* = 0.252, 95% CI: 0.222–0.283, *p* < 0.001; 2014: *β* = 0.246, 95% CI: 0.215–0.276, *p* < 0.001; 2018: *β* = 0.314, 95% CI: 0.274–0.355, *p* < 0.001) (Table 2).

#### 3.2.3. LGM With Time‐Invariant and Time‐Varying Variables

Two adjusted LGMs were estimated. Model E included all covariates except sleep quality, whereas Model F incorporated all covariates. According to the model fit indices, Model F demonstrated better fit than Model E.

The results of Model F indicated that better sleep quality was consistently associated with better psychological well‐being across all four survey waves. The β coefficients of sleep quality were 0.159 (95% CI: 0.122–0.197, *p* < 0.001), 0.193 (95% CI: 0.157–0.230, *p* < 0.001), 0.207 (95% CI: 0.170–0.244, *p* < 0.001), and 0.266 (95% CI: 0.227–0.305, *p* < 0.001) in 2008, 2011, 2014, and 2018, respectively.

### 3.3. Sensitivity Analysis

To ensure that the results were robust, three sensitivity tests were performed: (1) deleting all cases with missing values on any variables; (2) deleting cases with “unable to answer” for psychological well‐being; and (3) deleting cases with “unable to answer” for psychological well‐being and sleep quality. Similar to our primary results, sleep quality showed a positive association with psychological well‐being in both sensitivity tests (Supporting Tables S2 and S3).

In addition, subgroup analyses stratified by gender, education, and age yielded generally consistent findings (Supporting Table S4), suggesting that the observed longitudinal association between sleep quality and psychological well‐being was relatively stable across population subgroups.

## 4. Discussion

To the best of our knowledge, relatively few studies have examined the longitudinal association between sleep quality and psychological well‐being among Chinese older adults, and the temporal dynamics of this relationship remain insufficiently understood. This study found that both sleep quality and psychological well‐being showed a declining trend among older Chinese adults from 2008 to 2018. More importantly, the results indicate a stable longitudinal association between better sleep quality and higher psychological well‐being across the study period. These findings suggest that sleep quality and psychological well‐being are closely interconnected in later life; however, due to the observational nature of the data and the modeling strategy, causal inference cannot be established.

Consistent with previous studies conducted in China and other countries, declining psychological well‐being among older adults over time has also been reported in European and American populations [45, 46]. In the context of rapid population aging in China, psychological well‐being in later life has become an increasingly important public health concern. According to recent national demographic statistics, approximately 40 million individuals aged 80 years and above lived in China in 2024, accounting for about 2.8% of the total population [47]. This proportion is projected to increase substantially in the coming decades. Based on the medium‐scenario projections in the China Population Forecast Report 2023, the proportion of individuals aged 80 years and above is expected to reach 3.7% in 2030, 11.0% in 2050, 18.6% in 2070%, and 30.4% in 2100 [48]. These demographic trends highlight the growing importance of promoting psychological well‐being and healthy aging among the oldest‐old population in China.

This study also identified that psychological well‐being among older adults was associated with both time‐invariant and time‐varying factors, including education, marital status, income, health‐related behaviors (e.g., alcohol consumption and physical activity), sleep quality, ADL disability, and IADL disability. These findings are broadly consistent with previous research [49, 50]. For example, functional limitations in activities of daily living have been consistently associated with poorer mental health outcomes in older populations [50]. Older adults living with a spouse tend to report better mental health compared with those living alone or in other living arrangements [51]. In addition, alcohol consumption patterns and physical activity levels have been shown to be associated with psychological well‐being status. Notably, sleep quality was significantly associated with psychological well‐being in the present study, highlighting its relevance as an important correlate of psychological well‐being in later life.

Previous evidence has shown that sleep quality tends to decline with increasing age [52, 53], which is consistent with the findings of this study. Sleep problems are among the most prevalent health concerns in older populations worldwide [17]. A cohort study involving 2406 adults reported that reduced sleep efficiency was a major contributor to declining sleep quality, which is particularly common in older adults [54]. Epidemiological studies have also reported varying prevalence rates of poor sleep quality among older adults in different countries, including the United Kingdom, the United States, and Brazil [55–57], suggesting that sleep disturbance represents a global public health issue.

A growing body of literature has suggested that sleep quality is closely associated with emotional regulation, physical functioning, and overall well‐being in older adults. Poor sleep has been linked to adverse emotional states such as depressive symptoms, anxiety, and irritability [58]. For instance, irregular sleep patterns have been associated with an increased risk of depressive symptoms [59], and individuals with insomnia have been reported to exhibit a higher likelihood of anxiety and depression compared with those without insomnia [60]. In addition, poor sleep quality may be associated with reduced physical energy and functional capacity, which may further influence psychological well‐being [61]. Sleep disturbance has also been associated with hormonal dysregulation and increased risk of metabolic and cardiovascular conditions [62]. These findings suggest several potential pathways linking sleep and mental health; however, the present study cannot establish causal mechanisms due to its observational design.

Based on the observed longitudinal association between sleep quality and psychological well‐being, sleep health may represent a meaningful target for promoting healthy aging. Nonpharmacological interventions, such as cognitive behavioral therapy for insomnia (CBT‐I), sleep hygiene education, mindfulness‐based interventions, light therapy, and environmental modifications, have been widely recommended in previous studies [56, 63]. Pharmacological treatments, including melatonin and other sleep‐related medications, may be considered when clinically appropriate [24, 57].

At the behavioral level, promoting healthy lifestyle practices such as regular physical activity, smoking cessation, and reduced alcohol consumption may be beneficial for sleep health. Evidence from epidemiological and intervention studies suggests that lifestyle behaviors are closely associated with sleep quality in older adults, particularly physical activity and alcohol use patterns. Improvements in the sleep environment, including optimization of lighting, temperature, ventilation, and bedding comfort, may also support better sleep among older adults, consistent with environmental sleep research highlighting the role of bedroom and institutional settings in sleep regulation. In addition, strengthening family and social support may help reduce loneliness and improve overall sleep‐related well‐being, as social isolation has been identified as an important correlate of poor sleep in later life.

Notably, participants retained in the analytic sample were generally younger and healthier at baseline than those lost to follow‐up. Despite this relatively healthier profile, both sleep quality and psychological well‐being still showed declining trajectories over time. Significant longitudinal associations between sleep quality and psychological well‐being were still observed among these relatively healthier older adults, suggesting that sleep health may be important not only for highly vulnerable populations but also for community‐dwelling older adults with relatively preserved functional status. This finding may further suggest that declines in sleep quality and psychological well‐being could be even more pronounced among older adults with poorer baseline health conditions, greater functional impairment, or more advanced age, who were more likely to be lost during follow‐up. Therefore, the present findings may represent relatively conservative estimates of the longitudinal associations between sleep quality and psychological well‐being in the broader older population. These findings further highlight the importance of early sleep health promotion and psychological well‐being monitoring among community‐dwelling older adults before substantial functional decline occurs.

From a policy perspective, several strategies may be considered to support sleep health and psychological well‐being among older adults. First, sleep health screening could be integrated into routine geriatric assessments in community health centers, with established referral pathways for individuals with sleep problems. Such integrated care approaches are consistent with WHO recommendations for healthy aging and integrated primary care systems. Second, community‐based nonpharmacological sleep intervention programs, such as sleep hygiene education, relaxation training, and light therapy, could be developed and scaled up due to their low cost and high feasibility. CBT‐I, in particular, has been widely recommended as a first‐line nonpharmacological treatment for insomnia. Third, training programs for primary care providers could improve the early identification and management of sleep disturbances in older adults, consistent with evidence supporting task‐sharing approaches in primary care sleep management [64]. Fourth, sleep health promotion could be incorporated into national healthy aging strategies, in line with global healthy aging frameworks emphasizing functional ability and well‐being in older populations [65]. Finally, improvements in long‐term care environments, including noise reduction, lighting optimization, and bedding improvements, may contribute to more sleep‐friendly conditions for older populations, consistent with evidence on institutional sleep environment modification [64].

## 5. Limitations

There are some limitations to this study. First, substantial sample attrition occurred during the long follow‐up period, mainly due to mortality and loss to follow‐up among older adults. Baseline comparisons indicated that participants retained in the analytic sample were generally younger and healthier than those lost to follow‐up, suggesting the possibility of survivor bias and selective attrition. In addition, relatively high baseline missingness in mental health and chronic disease variables among participants lost to follow‐up suggests that attrition may not have occurred completely at random. Although such attrition patterns are common in longitudinal aging studies, the potential influence of selection bias on the estimated longitudinal associations cannot be completely excluded. Therefore, the findings should be interpreted with caution when generalizing to older populations with poorer baseline health conditions. Second, sleep quality was assessed using a single self‐rated item rather than a multidimensional validated sleep scale such as the PSQI. Although this measure has been widely used in population‐based surveys and large epidemiological studies involving older adults, it may not fully capture all dimensions of sleep quality. Third, although LGM was useful for examining longitudinal trajectories and associations over time, the observational design of the study does not permit definitive causal inference. Residual confounding and potential bidirectional relationships between sleep quality and psychological well‐being may still exist despite adjustment for multiple covariates.

## 6. Conclusion

In this study, we examined the longitudinal association between sleep quality and psychological well‐being based on the CLHLS database from 2008 to 2018. This study confirmed that sleep quality had a positive longitudinal association with psychological well‐being, suggesting potential pathways for improving older adults’ psychological well‐being. Simultaneously, while implementing scientific and efficient measures aimed at ensuring psychological well‐being among older adults, additional focus should be given to the target groups, such as those with less education, no spouse, lower income, physical inactivity, and disabilities.

## Author Contributions

Yunyun Huang and Lizheng Ge drafted the manuscript, designed the study, and conducted the literature review. Songjia Zhang, Rujia Zhang, Xiaoyi Wang, and Qingren Yang collected the literature. Chun Chen, Xiangyang Zhang, Xuelan Zhao, and Jiajia Liu designed the original version of the plans.

## Funding

This work was supported by National Natural Science Foundation of China (72274141).

## Disclosure

All authors read and approved the final manuscript.

## Ethics Statement

All procedures in this study were carried out in accordance with the ethical standards of the institutional and/or national research committee and with the 1964 Helsinki Declaration and its later amendments or comparable ethical standards. Written informed consent was obtained from all participants and/or their proxy respondents, and the study was approved by the Research Ethics Committee of Peking University (IRB00001052–13074). All the respondents offered a written consent before participating in the survey. Informed consent was obtained from all subjects and/or their legal guardian(s).

## Consent

Please see the Ethics Statement.

## Conflicts of Interest

The authors declare no conflicts of interest.

## Supporting Information

Additional supporting information can be found online in the Supporting Information section.

## Supporting information


**Supporting Information** Supporting Table S1. Baseline characteristics (2008) of participants retained in the analytic sample versus those lost to follow‐up among adults aged ≥ 65 years. Supporting Table S2. Comparison of the final LGM estimated using multiple imputation analysis and complete‐case analysis. Supporting Table S3. Sensitive analysis of this study. Supporting Table S4. Subgroup analysis of this study.

## Data Availability

The data that support the findings of this study are openly available in Peking University Open Research Data at https://opendata.pku.edu.cn/dataverse/CHADS;jsessionid%3D13ff7a47a67350bee6776fe1ede2.

## References

[1] Hale L. , Troxel W. , and Buysse D. J. , Sleep Health: An Opportunity for Public Health to Address Health Equity, Annual Review of Public Health. (2020) 41, no. 1, 81–99, 10.1146/annurev-publhealth-040119-094412. PMC7944938 31900098

[2] Patel V. , Saxena S. , Lund C. et al., The Lancet Commission on Global Mental Health and Sustainable Development, Lancet. (2018) 392, no. 10157, 1553–1598, 10.1016/s0140-6736(18)31612-x. 30314863

[3] World Health Organization , World Mental Health Report: Transforming Mental Health for all, 2022, World Health Organization, Geneva.

[4] World Health Organization , Mental Health of Older Adults, 2025, WHO, Geneva, https://www.who.int/news-room/fact-sheets/detail/mental-health-of-older-adults.

[5] Chai K. C. , Zhang Y. B. , Chang K. C. et al., The Influence of Social and Commercial Pension Insurance Differences and Social Capital on the Mental Health of Older adults-Microdata from China, Frontiers in Public Health. (2022) 10, 10.3389/fpubh.2022.1005257. PMC9683030 36438206

[6] Li K. , He Y. , Li X. et al., Spatiotemporal Evolution of the Prevalence of Depressive Symptoms Among Older Adults-China, 2013-2020, China CDC Weekly. (2025) 7, no. 21, 732–736. 40620850 10.46234/ccdcw2025.120 PMC12227995

[7] Fan Y. , Wu J. , Liu Z. et al., Global Burden of Mental Disorders in 204 Countries and Territories, 1990-2021: Results from the Global Burden of Disease Study 2021, BMC Psychiatry. (2025) 25, no. 1, 10.1186/s12888-025-06932-y. PMC12080068 40375174

[8] Christensen M. K. , Lim C. C. W. , Saha S. et al., The Cost of Mental Disorders: a Systematic Review, Epidemiology and Psychiatric Sciences. (2020) 29, 10.1017/s204579602000075x. PMC7443800 32807256

[9] Ryff C. D. and Keyes C. L. , The Structure of Psychological well-being Revisited, Journal of Personality and Social Psychology. (1995) 69, no. 4, 719–727, 10.1037/0022-3514.69.4.719. 7473027

[10] Menassa M. , Stronks K. , Khatmi F. et al., Concepts and Definitions of Healthy Ageing: A Systematic Review and Synthesis of Theoretical Models, EClinicalMedicine. (2023) 56, 10.1016/j.eclinm.2022.101821. PMC9852292 36684393

[11] Kohn J. N. , Pugh E. , Wang Y. et al., Trends, Heterogeneity, and Correlates of Mental Health and Psychosocial well-being in later-life: Study of 590 community-dwelling Adults Aged 40-104 Years, Aging & Mental Health. (2023) 27, no. 6, 1198–1207, 10.1080/13607863.2022.2078790. 35622016

[12] Giebel C. , Gabbay M. , Shrestha N. et al., Community-Based Mental Health and well-being Interventions for Older Adults in low-and middle-income Countries: A Systematic Review and meta-analysis, BMC Geriatrics. (2022) 22, no. 1, 10.1186/s12877-022-03453-1. PMC9520120 36175867

[13] Muzni K. , Dijk J. A. G. D. , and Lazar A. S. , Self-Reported Sleep Quality is More Closely Associated With Mental and Physical Health than Chronotype and Sleep Duration in Young Adults: A Multi-Instrument Analysis, Journal of Sleep Research. (2021) 30, no. 1, 10.1111/jsr.13152. PMC11475679 32783404

[14] Kerkhof G. A. , Epidemiology of Sleep and Sleep Disorders in the Netherlands, Sleep Medicine. (2017) 30, 229–239, 10.1016/j.sleep.2016.09.015. 28215254

[15] China Sleep Research Society , China’s National Healthy Sleep in 2022, 2022.

[16] Ou K. L. , Chen Y. Q. , Wang Y. et al., Effect of Square Dance Interventions on Physical and Mental Health Among Chinese Older Adults: A Systematic Review, International Journal of Environmental Research and Public Health. (2022) 19, no. 10, 10.3390/ijerph19106181. PMC9141523 35627716

[17] Marini C. M. , Wilson S. J. , Nah S. et al., Rumination and Sleep Quality Among Older Adults: Examining the Role of Social Support, The Journals of Gerontology: Psychological Science. (2021) 76, no. 10, 1948–1959, 10.1093/geronb/gbaa230. PMC8598998 33378473

[18] Hepsomali P. and Groeger J. A. , Diet, Sleep, and Mental Health: Insights from the UK Biobank Study, Nutrients. (2021) 13, no. 8, 10.3390/nu13082573. PMC8398967 34444731

[19] Gao Q. , Xu Q. , Guo X. et al., Particulate Matter Air Pollution Associated with Hospital Admissions for Mental Disorders: A time-series Study in Beijing, China, European Psychiatry. (2017) 44, 68–75, 10.1016/j.eurpsy.2017.02.492. 28545011

[20] Sarkar C. , Webster C. , and Gallacher J. , Residential Greenness and Prevalence of Major Depressive Disorders: A cross-sectional, Observational, Associational Study of 94 879 Adult UK Biobank Participants, The Lancet Planetary Health. (2018) 2, no. 4, e162–e173, 10.1016/s2542-5196(18)30051-2. 29615217

[21] Kowall S. M. , Gallant K. A. , Graham S. A. et al., Sleep Disturbance During COVID-19: Correlates and Predictive Ability for Mental Health Symptomatology in a Canadian Online Sample, General Hospital Psychiatry. (2023) 80, 48–53, 10.1016/j.genhosppsych.2023.01.002. 36638699 PMC9816073

[22] Lu J. and Liu K. , Sleep Quality and Mental Health of the Elderly in China: Evidence from Longitudinal Data, Sleep quality and mental health of the elderly in China: Evidence from Longitudinal Data. China Population and Development Studies. (2021) 5, no. 4, 378–393, 10.1007/s42379-021-00096-4.

[23] Fitzmaurice G. M. , Laird N. M. , and Ware J. H. , Applied Longitudinal Analysis, 2nd Edition, 2011, Wiley Series in Probability and Statistics.

[24] Kothari V. , Kothari P. , Goyal S. , Anothaisintawee T. , and Reutrakul S. , Sleep Interventions and Glucose Metabolism: Systematic Review and meta-analysis, Sleep Medicine. (2021) 78, 24–35, 10.1016/j.sleep.2020.11.035. 33383394

[25] Bighelli I. , Rodolico A. , García-Mieres H. et al., Psychosocial and Psychological Interventions for Relapse Prevention in Schizophrenia: A Systematic Review and Network meta-analysis, The Lancet Psychiatry. (2021) 8, no. 11, 969–980, 10.1016/s2215-0366(21)00243-1. 34653393

[26] Le L. K. D. , Esturas A. C. , Mihalopoulos C. et al., Cost-Effectiveness Evidence of Mental Health Prevention and Promotion Interventions: A Systematic Review of Economic Evaluations, PLoS Medicine. (2021) 18, no. 5, 10.1371/journal.pmed.1003606. PMC8148329 33974641

[27] Zhou Z. , Mao F. , Zhang W. et al., A Longitudinal Analysis of the Association Between the Living Arrangements and Psychological well-being of Older Chinese Adults: The Role of Income Sources, BMC Geriatrics. (2019) 19, no. 1, 10.1186/s12877-019-1371-0. PMC6904999 31822282

[28] Zhang J. , Li L. W. , and McLaughlin S. J. , Psychological Well-Being and Cognitive Function Among Older Adults in China: A Population-Based Longitudinal Study, Journal of Aging and Health. (2022) 34, no. 2, 173–183, 10.1177/08982643211036226. 34510952

[29] Fabbri M. , Beracci A. , Martoni M. et al., Measuring Subjective Sleep Quality: A Review, International Journal of Environmental Research and Public Health. (2021) 18, no. 3, 10.3390/ijerph18031082. PMC7908437 33530453

[30] Buysse D. J. , Reynolds C. F. , Monk T. H. et al., The Pittsburgh Sleep Quality Index: A New Instrument for Psychiatric Practice and Research, Psychiatry Research. (1989) 28, no. 2, 193–213, 10.1016/0165-1781(89)90047-4. 2748771

[31] Liu N. , Wang Y. , Chen X. et al., Association of Leisure Activities with Sleep Duration and Quality in Chinese Older Adults, Sage Open. (2024) 14.

[32] Gu D. , Sautter J. , Pipkin R. et al., Sociodemographic and Health Correlates of Sleep Quality and Duration Among Very Old Chinese, Sleep. (2010) 33, no. 5, 601–610, 10.1093/sleep/33.5.601. 20469802 PMC2864875

[33] Menezes-Júnior L. , Psychometric Properties of the two-item Pittsburgh Sleep Quality Index (PSQI-2) in a Cohort of community-dwelling Older Men: The Mros Sleep Study, Biological Timing and Sleep. (2026) 3, no. 1, 10.1038/s44323-025-00069-7. PMC12972079 41803458

[34] Onen F. , Moreau T. , Gooneratne N. S. et al., A Three-Item Instrument for Measuring Daytime Sleepiness: The Observation and Interview Based Diurnal Sleepiness Inventory (ODSI), Journal of Clinical Sleep Medicine. (2016) 12, no. 4, 505–512, 10.5664/jcsm.5676. 26612511 PMC4795276

[35] Ren T. , Yu X. , and Yang W. , Do Cognitive and Non-cognitive Abilities Mediate the Relationship Between Air Pollution Exposure and Mental Health?, PLoS One. (2019) 14, no. 10, 10.1371/journal.pone.0223353. PMC6808496 31644533

[36] Walsh J. L. , Senn T. E. , and Carey M. P. , Longitudinal Associations Between Health Behaviors and Mental Health in low-income Adults, Translational Behavioral Medicine. (2013) 3, no. 1, 104–113, 10.1007/s13142-012-0189-5. 23997836 PMC3717991

[37] Duncan T. E. and Duncan S. C. , An Introduction to Latent Growth Curve Modeling, Behavior Therapy. (2004) 35, no. 2, 333–363, 10.1016/s0005-7894(04)80042-x.

[38] Graham J. W. , Olchowski A. E. , and Gilreath T. D. , How Many Imputations are Really Needed? Some Practical Clarifications of Multiple Imputation Theory, Prevention Science. (2007) 8, no. 3, 206–213, 10.1007/s11121-007-0070-9. 17549635

[39] Bodner T. E. , What Improves with Increased Missing Data Imputations?, Structural Equation Modeling: A Multidisciplinary Journal. (2008) 15, no. 4, 651–675, 10.1080/10705510802339072.

[40] Rubin D. B. , Multiple Imputation for Nonresponse in Surveys, 1987, John Wiley & Sons, New York.

[41] Cui S. , Yu Y. , Dong W. et al., Are There Gender Differences in the Trajectories of self-rated Health Among Chinese Older Adults? an Analysis of the Chinese Longitudinal Healthy Longevity Survey (CLHLS), BMC Geriatrics. (2021) 21, no. 1, 10.1186/s12877-021-02484-4. PMC8522225 34663221

[42] Yu Y. , Yang X. , Wang Y. et al., Longitudinal Association Between Home and Community-based Services Provision and Cognitive Function in Chinese Older Adults: Evidence from the Chinese Longitudinal Healthy Longevity Survey, Health and Social Care in the Community. (2021) 29, no. 6, e288–e298. 33761178 10.1111/hsc.13353

[43] Zeng Y. , Feng Q. , Hesketh T. et al., Survival, Disabilities in Activities of Daily Living, and Physical and Cognitive Functioning Among the oldest-old in China: A Cohort Study, Lancet. (2017) 389, no. 10079, 1619–1629, 10.1016/s0140-6736(17)30548-2. 28285816 PMC5406246

[44] Weuve J. , Tchetgen Tchetgen E. J. , Glymour M. M. et al., Accounting for Bias due to Selective Attrition: The Example of Smoking and Cognitive Decline, Epidemiology. (2012) 23, no. 1, 119–128, 10.1097/ede.0b013e318230e861. 21989136 PMC3237815

[45] Lüdecke D. and von dem Knesebeck O. , Decline in Mental Health in the Beginning of the COVID-19 Outbreak Among European Older Adults-Associations with Social Factors, Infection Rates, and Government Response, Frontiers in Public Health. (2022) 10, 10.3389/fpubh.2022.844560. PMC8963994 35359766

[46] Jain S. , Murphy T. E. , O’Leary J. R. et al., Association Between Socioeconomic Disadvantage and Decline in Function, Cognition, and Mental Health After Critical Illness Among Older Adults: A Cohort Study, Annals of Internal Medicine. (2022) 175, no. 5, 644–655, 10.7326/m21-3086. 35254879 PMC9316386

[47] National Bureau of Statistics of China , Statistical Communiqué of the People’s Republic of China on the 2024 National Economic and Social Development, 2025, National Bureau of Statistics of China, Beijing.

[48] Chinese Population and Development Research Center , China Population Forecast Report 2023, 2023, China Population Publishing House, Beijing.

[49] Wei K. , Liu Y. , Yang Z. et al., Living Arrangement Modifies the Associations of Loneliness With Adverse Health Outcomes in Older Adults: Evidence from the CLHLS, BMC Geriatrics. (2022) 22, no. 1, 10.1186/s12877-021-02742-5. PMC8764854 35038986

[50] Liu Y. B. , Liu L. , Li Y. F. et al., Health Literacy, Physical and Mental Health, and Activities of Daily Living Among Older Chinese Adults in Nursing Homes, Asia-Pacific Journal of Public Health. (2018) 30, no. 6, 592–599, 10.1177/1010539518800368. 30324821

[51] Lee K. and Marier P. , Aging in Place with a Spouse in Need: Neighborhood Cohesion and Older Adult Spouses’ Physical and Mental Health, International Journal of Aging and Human Development. (2021) 93, no. 4, 1012–1030, 10.1177/0091415020974616. 33241942

[52] Kim M. , Seol J. , Sato T. et al., Effect of 12-Week Intake of Nicotinamide Mononucleotide on Sleep Quality, Fatigue, and Physical Performance in Older Japanese Adults: A Randomized, Double-Blind Placebo-Controlled Study, Nutrients. (2022) 14, no. 4, 10.3390/nu14040755. PMC8877443 35215405

[53] Chaput J. P. , Dutil C. , and Sampasa-Kanyinga H. , Sleeping Hours: What is the Ideal Number and How Does Age Impact This?, Nature and Science of Sleep. (2018) 10, 421–430, 10.2147/nss.s163071. PMC6267703 30568521

[54] Gadie A. , Shafto M. , Leng Y. et al., How are age-related Differences in Sleep Quality Associated with Health Outcomes? an Epidemiological Investigation in a UK Cohort of 2406 Adults, BMJ Open. (2017) 7, no. 7, 10.1136/bmjopen-2016-014920. PMC5642766 28760786

[55] Didikoglu A. , Maharani A. , Payton A. et al., Longitudinal Sleep Efficiency in the Elderly and its Association with Health, Journal of Sleep Research. (2020) 29, no. 3, 10.1111/jsr.12898. 31313420

[56] Khatib H. K. A. , Hall W. L. , Creedon A. et al., Sleep Extension is a Feasible Lifestyle Intervention in free-living Adults Who are Habitually Short Sleepers: A Potential Strategy for Decreasing Intake of Free Sugars? A Randomized Controlled Pilot Study, The American Journal of Clinical Nutrition. (2018) 107, no. 1, 43–53, 10.1093/ajcn/nqx030. 29381788 PMC5972593

[57] Murawski B. , Wade L. , Plotnikoff R. C. et al., A Systematic Review and meta-analysis of Cognitive and Behavioral Interventions to Improve Sleep Health in Adults Without Sleep Disorders, Sleep Medicine Reviews. (2018) 40, 160–169, 10.1016/j.smrv.2017.12.003. 29397329

[58] Liu X. , Liu C. , Tian X. et al., Sleep Quality, Depression and Frailty Among Chinese community-dwelling Older Adults, Geriatric Nursing. (2021) 42, no. 3, 714–720, 10.1016/j.gerinurse.2021.02.020. 33836251

[59] Fang Y. , Forger D. B. , Frank E. et al., Day-To-Day Variability in Sleep Parameters and Depression Risk: A Prospective Cohort Study of Training Physicians, Npj Digital Medicine. (2021) 4, no. 1, 10.1038/s41746-021-00400-z. PMC7892862 33603132

[60] Taylor D. J. , Lichstein K. L. , Durrence H. H. et al., Epidemiology of Insomnia, Depression, and Anxiety, Sleep. (2005) 28, no. 11, 1457–1464, 10.1093/sleep/28.11.1457. 16335332

[61] Denison H. J. , Jameson K. A. , Sayer A. A. et al., Poor Sleep Quality and Physical Performance in Older Adults, Sleep Health. (2021) 7, no. 2, 205–211, 10.1016/j.sleh.2020.10.002. 33223446

[62] Centers for Disease Control and Prevention , Sleep and Sleep Disorders, https://www.cdc.gov/sleep/index.html.

[63] Jun J. , Kapella M. C. , and Hershberger P. E. , Non-Pharmacological Sleep Interventions for Adult Patients in Intensive Care Units: A Systematic Review, Intensive and Critical Care Nursing. (2021) 67, 10.1016/j.iccn.2021.103124. 34456110

[64] Espie C. A. , Kyle S. D. , Williams C. et al., A Randomized, placebo-controlled Trial of Online Cognitive Behavioral Therapy for Chronic Insomnia Disorder Delivered via an Automated media-rich Web Application, Sleep. (2012) 35, no. 6, 769–781, 10.5665/sleep.1872. 22654196 PMC3353040

[65] World Health Organization , Decade of Healthy Ageing 2021–2030, 2020, World Health Organization, Geneva.

