# Supplementary material for: Better Sleep Better Psychological Well‐Being Among Older Adults: Evidence From the Chinese Longitudinal Healthy Longevity Survey (CLHLS)
Source: J Aging Res. 2026 Jul 29;2026:1858442. doi: 10.1155/jare/1858442 (PMC13420322; doi:10.1155/jare/1858442)
Supplement: Supplementary file 1 — Supporting Information Supporting Table S1. Baseline characteristics (2008) of participants retained in the analytic sample versus those lost to follow‐up among adults aged ≥ 65 years. Supporting Table S2. Comparison of the final LGM estimated using multiple imputation analysis and complete‐case analysis. Supporting Table S3. Sensitive analysis of this study. Supporting Table S4. Subgroup analysis of this study. [file JARE-2026-1858442-s001.docx]

**Supplementary Table S1.** Baseline characteristics (2008) of participants retained in the analytic sample versus those lost to follow-up among adults aged ≥65 years.

| **Baseline characteristic** | **Analytic sample (n=2,371)** | **Attrition sample (n=14,196)** | ***t/χ2*** | ***P*** | |  |
| --- | --- | --- | --- | --- | --- | --- |
| **Age** | 75.83±8.11 | 89.47±10.57 | -72.305 | <0.001 | |  |
| **gender** | | | | | |  |
| Woman | 1 280 (53.99%) | 8 298 (58.45%) | 16.625 | <0.001 | |  |
| Man | 1 091 (46.01%) | 5 898 (41.55%) |  |  | |  |
| **education** | | | | | |  |
| Illiteracy | 1 161 (48.97%) | 9 305 (65.55%) | 243.093 | <0.001 | |  |
| Primary school or above | 1 205 (50.82) % | 4 846 (34.14%) |  |  | |  |
| Missing | 5 (0.21%) | 45 (0.32%) |  |  | |  |
| **Marital status** | | | | | |  |
| Unmarried or widowed | 938 (39.56%) | 10 435 (73.51%) | 1 087.734 | <0.001 | |  |
| married | 1 433 (60.44%) | 3 761 (26.49%) |  |  | |  |
| Missing | 0 (0) | 0 (0) |  |  | |  |
| **Residence** | | | | | |  |
| Urban | 304 (12.82%) | 3 022 (21.29%) | 93.972 | <0.001 | |  |
| Town | 477 (20.12%) | 2 778 (19.57%) |  |  | |  |
| Rural | 1 590 (67.06%) | 8 396 (59.14%) |  |  | |  |
| **Living with family member** | | | | |  |  |
| No | 391 (16.49%) | 2 464 (17.36%) | 1.068 | 0.301 | |  |
| Yes | 1 980 (83.51%) | 11 732 (82.64%) |  |  | |  |
| Missing | 0 (0) | 0 (0) |  |  | |  |
| **Income** | | | | | |  |
| Very poor | 56 (2.36%) | 470 (3.31%) | 13.664 | 0.008 | |  |
| poor | 324 (13.67%) | 2 165 (15.25%) |  |  | |  |
| Fair | 1 689 (71.24%) | 9 628 (67.82%) |  |  | |  |
| Good | 273 (11.51%) | 1 747 (12.31%) |  |  | |  |
| Very good | 24 (1.01%) | 143 (1.01%) |  |  | |  |
| Missing | 5 (0.21%) | 43 (0.30%) |  |  | |  |
| **Sleep quality** | | | | | |  |
| Very poor | 23 (0.97%) | 110 (0.77%) | 20.302 | <0.001 | |  |
| poor | 247 (10.42%) | 1 223 (8.62%) |  |  | |  |
| Fair | 574 (24.21%) | 3 565 (25.11%) |  |  | |  |
| Good | 1 167 (49.22%) | 7 383 (52.01%) |  |  | |  |
| Very good | 358 (15.10%) | 1 814 (12.78%) |  |  | |  |
| Missing | 2 (0.08%) | 101 (0.71%) |  |  | |  |
| **Physical activity** | | | | | | |
| No | 1 540 (64.95%) | 10 489 (73.89%) | 81.664 | <0.001 | |  |
| Yes | 831 (35.05%) | 3 706 (26.11%) |  |  | |  |
| Missing | 0 (0) | 1 (0.01%) |  |  | |  |
| **Drinking** | | | | | |  |
| No | 1 863 (78.57%) | 11 915 (83.93%) | 41.654 | <0.001 | |  |
| Yes | 508 (21.43%) | 2 281 (16.07%) |  |  | |  |
| Missing | 0 (0) | 0 (0) |  |  | |  |
| **Smoking** | | | | | |  |
| No | 1 850 (78.03%) | 11 899 (83.82%) | 48.303 | <0.001 | |  |
| Yes | 521 (21.97%) | 2 297 (16.18%) |  |  | |  |
| Missing | 0 (0) | 0 (0) |  |  | |  |
| **ADL disability** | | | | | | |
| No | 2 314 (97.60%) | 10 521 (74.11%) | 641.796 | <0.001 | |  |
| Yes | 57 (2.40%) | 3 674 (25.88%) |  |  | |  |
| Missing | 0 (0) | 1 (0.01%) |  |  | |  |
| **IADL disability** | | | | | | |
| No | 1 624 (68.49%) | 3 386 (23.85%) | 1919.358 | <0.001 | |  |
| Yes | 747 (31.51%) | 10 810 (76.15%) |  |  | |  |
| Missing | 0 (0) | 0 (0) |  |  | |  |
| **Chronic disease** | | | | |  |  |
| 0 | 1 026 (43.27%) | 2 547 (17.94%) | 1303.081 | <0.001 | |  |
| 1 | 1 304 (55.00%) | 7 932 (55.87%) |  |  | |  |
| Missing | 41 (1.73%) | 3 717 (26.18%) |  |  | |  |
| **Psychological Well-being** | 26.26±3.73 | 24.93±4.04 | 15.382 | <0.001 | |  |
| Missing | 92 (3.88%) | 3004 (21.16%) |  |  | |  |
| *Note:* The analytic sample included participants who completed all four waves of the survey. Individuals aged below 65 years at baseline (2008) were excluded from the final analysis. Comparisons were conducted between the retained analytic sample and those lost to follow-up. | | | | | |  |

**Supplementary Table S2.** Comparison of the final LGM estimated using multiple imputation analysis and complete-case analysis

| Year | Multiple imputed analysis | | Complete-case analysis | |
| --- | --- | --- | --- | --- |
|  | *β* (95% CI) | *P* | *β* (95% CI) | *P* |
| 2008 | 0.159 (0.122~0.297) | <0.001 | 0.179 (0.124~0.233) | <0.001 |
| 2011 | 0.193 (0.157~0.230) | <0.001 | 0.195 (0.144~0.247) | <0.001 |
| 2014 | 0.207 (0.170~0.244) | <0.001 | 0.222 (0.171~0.273) | <0.001 |
| 2018 | 0.266 (0.227~0.305) | <0.001 | 0.307 (0.259~0.356) | <0.001 |

**Supplementary Table S3.** Sensitive analysis of this study

|  | **Sensitivity analysis A** | | **Sensitivity analysis B** | |
| --- | --- | --- | --- | --- |
|  | β (95 %CI) | P-value | β (95 %CI) | P-value |
| 2008 | 0.167(0.122~0.212) | <0.001 | 0.164(0.118~0.210) | <0.001 |
| 2011 | 0.202(0.159~0.245) | <0.001 | 0.203(0.160~0.246) | <0.001 |
| 2014 | 0.211(0.168~0.254) | <0.001 | 0.211(0.168~0.254) | <0.001 |
| 2018 | 0.280(0.238~0.321) | <0.001 | 0.281(0.240~0.323) | <0.001 |
| *Note:* Sensitivity analysis A: dropping psychological well-being cases who with “unable to answer”; Sensitivity analysis B: dropping self-rated sleep quality and psychological well-being cases who with “unable to answer”. | | | | |

**Supplementary Table S4.** Subgroup analysis of this study

| **Model F** | **Gender** | | **Education** | | **Age** | |
| --- | --- | --- | --- | --- | --- | --- |
|  | **Women** | **Men** | **Illiterate** | **Primary school and above** | **Age at baseline < 80** | Age at baseline ≥ 80 |
| 0.156 (0.119-0.194)^***^ | 0.143 (0.091-0.195)^***^ | 0.181 (0.126-0.236)^***^ | 0.127 (0.070-0.184)^***^ | 0.194 (0.073-0.181)^***^ | 0.180 (0.134-0.225)^***^ | 0.126 (0.058-0.193)^***^ |
| 0.195 (0.159-0.231)^***^ | 0.198 (0.148-0.247)^***^ | 0.193 (0.139-0.247)^***^ | 0.173 (0.117-0.223)^***^ | 0.222 (0.118-0.227)^***^ | 0.200 (0.158-0.243)^***^ | 0.168 (0.105-0.232)^***^ |
| 0.214 (0.178-0.251)^***^ | 0.250 (0.197-0.303)^***^ | 0.152 (0.098-0.206)^***^ | 0.249 (0.186-0.294)^***^ | 0.173 (0.192-0.305)^***^ | 0.227 (0.182-0.272)^***^ | 0.184 (0.112-0.255)^***^ |
| 0.262 (0.225-0.300)^***^ | 0.305 (0.257-0.353)^***^ | 0.212 (0.157-0.267)^***^ | 0.324 (0.256-0.358)^***^ | 0.231 (0.263-0.385)^***^ | 0.274 (0.225-0.323)^***^ | 0.259 (0.190-0.329)^***^ |
| *Note:* Standardized β (95% CI) in table above. Model F is the adjusted model with all covariates including self-rated sleep quality.  *p < 0.05, **p < 0.01, ***p < 0.001. | | | | | | |
